# Conflict-attributable mortality in Tigray Region, Ethiopia: Evidence from a survey of the Tigrayan diaspora

**DOI:** 10.1186/s12963-025-00380-2

**Published:** 2025-05-22

**Authors:** Catherine R. McGowan, Sarah A. Cassidy-Seyoum, Promise Ekoriko, Mervat Alhaffar, Lucia Cassini, Jennifer Palmer, Abdihamid Warsame, Francesco Checchi

**Affiliations:** 1 Department of Infectious Disease Epidemiology and International Health, Faculty of Epidemiology and Population Health, London School of Hygiene and Tropical Medicine, Keppel Street, London, WC1E 7HT UK; 2 Global Tropical Health Division, Menzies School of Health Research, Royal Darwin Hospital Campus, Tiwi, Australia; 3 Information Technology Services, London School of Hygiene and Tropical Medicine, Keppel Street, London, WC1E 7HT UK; 4 Department of Global Health and Development, Faculty of Public Health and Policy, 15-17 Tavistock Place, London, WC1H 9SH UK

**Keywords:** Respondent-driven sampling, Mortality estimation, WebRDS, Conflict, Tigray, Diaspora

## Abstract

**Background:**

The war in Tigray Region, Ethiopia (November 2020 to November 2022) ended with formal commitments to accountability, but these have yet to produce publicly available accounts of the harms caused by the conflict.

**Methods:**

We carried out an online survey of the Tigrayan diaspora to estimate mortality amongst children, adults, and older adults during, and prior to, the war-period. We collected retrospective demographic information on respondents’, and their spouses’, extended family inside Tigray. To mitigate selection bias, we standardised mortality estimates by rural-urban residence and wealth index.

**Results:**

Of 1011 participant-reported decedents, 810 died within Tigray, and 310 of these individuals died during the war-period. Of the 310 deaths in Tigray during the war-period, 224 (72.3%) died from intentional injuries. The standardised mortality rate for adults (15–49 years) was 21.3 per 1000 person-years (29.4 for men, 14.8 for women) during the war, and 1.0 in the preceding period (2010–2020). The mortality rate amongst older adults (≥ 60 years) was 45.1 per 1000 person-years during the war-period, compared to 22.8 in 2010–2020, and is higher than the period encompassing the Ethiopian Civil War (1974–1991) and Tigray/Wollo Famine (1984–1985). The mortality ratio (men to women) was approximately 2:1 in both adults, and older adults. The mortality rate amongst adults and older adults had been declining across the pre-war periods. Few deaths were reported amongst children. We estimate that the conflict has resulted in more than 102,000 deaths amongst those aged ≥ 15 years.

**Conclusions:**

Our study suggests a significant elevation in all-cause mortality, largely driven by intentional injuries. Although our pre-war-period data are likely under-reported, comparisons with other pre-war estimates corroborate these plausible elevations, particularly amongst adults. The mortality ratio, when compared to those from other settings, does not support assertions that the military strategy primarily involved the targeting of adult males, and instead suggests widespread killing of women and older adults who would not have posed a combat threat.

**Supplementary Information:**

The online version contains supplementary material available at 10.1186/s12963-025-00380-2.

## Background

On 2 November 2022 - amid reports of war crimes, ethnic cleansing, and widespread human rights abuses [1, 2, 3] - the Ethiopian Government and the Tigray People’s Liberation Front (TPLF) agreed to end hostilities following two years of civil war. Despite a formal commitment to peace, the two years since the signing of the peace agreement have been tarnished by ongoing reports of human rights violations [1, 3, 4] and growing concerns about a resumption of the conflict [5, 6].

Quantifying the impact of the conflict has been hampered by lack of access - particularly in Western Tigray and areas bordering Eritrea which have experienced some of the most intense attacks on civilians - and frequent telecommunications/internet disruptions [2]. Journalists reporting on the conflict have been denied access to conflict-affected regions; some have been arrested [1]. In late 2021 the UN Human Rights Council (UNHRC) established the International Commission of Human Rights Experts on Ethiopia (ICHREE) to investigate potential violations of international law; however, in October 2023 the UNHRC chose not to renew ICHREE’s mandate [1]. Media reporting has largely focussed on specific attacks on civilians including those in Central and Western Tigray [7, 8], and in villages bordering Eritrea [9, 10, 11]. Whilst these reports evidence the brutality of the conflict, they paint an incomplete picture of the conflict as a whole as the impact of a conflict cannot be measured solely in terms of its most proximate effects, i.e. fatal injuries [12]. Often the indirect impacts - including malnutrition (and associated sequelae), deaths resulting from lack of access to health services (e.g. routine childhood vaccination, maternal and newborn health) - far outweigh the number of casualties due to violent injury [13]. Though these downstream health impacts are typically life limiting, they are difficult to measure and often are not considered when evidencing violations of international law [13, 14, 15]. The paucity of available information about the impact of the Tigray conflict may have contributed to the diminishing international interest in what has been described as a ‘forgotten’ war [2, 3, 16].

Population mortality is an objective indicator of the scale and severity of a conflict [13]. Several estimates of mortality during the Tigray conflict have been published, including cause-specific mortality [17, 18, 19], the under five mortality ratio (U5MR) [20], and maternal [21] and neonatal mortality ratios [22]. Taken together these estimates suggest that the conflict in Tigray has resulted in substantial loss of life; however, each estimate is subject to important limitations, and none provides all-cause mortality across the entire region.

To fill this gap, whilst circumventing access constraints, we surveyed the global Tigrayan diaspora to estimate all-cause mortality estimates for children, adults, and older adults for the entire region of Tigray before, and subsequent to, the start of the conflict on 4 November 2020.

## Methods

### Study population and sampling process

We used a web-based respondent-driven sampling (webRDS) approach to disseminate a mortality survey amongst members of the global Tigrayan diaspora. Respondent-driven sampling (RDS) is a method of chain-referral recruitment that occurs within peer networks, outside the direct influence of the research team [23]. The recruitment process involves the recruitment of ‘seeds’ (i.e. well-networked members of the study population) who are responsible for initiating the survey cascade within their networks. Web-based RDS (webRDS) offers several advantages over RDS [24] and can be designed to disseminate a survey anonymously. We describe elsewhere the design and development of our webRDS solution, and its use in estimating conflict-attributable mortality in Yemen [25, 26].

A member of the study team reached out to diaspora organisations and key contacts within the Tigrayan diaspora to discuss participating in the study as a seed. Participating seeds were sent an invitation (via email, WhatsApp, or SMS) to complete the mortality survey in ODK (GetODK. 2025. San Diego, CA: https://getodk.org/). Eligibility was determined, and consent obtained, at the beginning of the mortality survey. Respondents were eligible if they: were of Tigrayan origin, were between 18 and 49 years of age, lived outside of Tigray, and were confident that no other close family member had completed the mortality survey. The webRDS web-application allowed respondents to invite up to five individuals within their networks. Information about the study was disseminated via social media platforms and through online information sessions. We collected data over 23 weeks from 6 November 2022 (immediately following the Cessation of Hostilities Agreement) to 18 April 2023.

Survey responses were anonymous; the only information collected about respondents themselves was: current residence (Tigray/outside Tigray), marital status (to determine if they were eligible to complete the survey on behalf of a spouse), and place of birth (Tigray/outside Tigray). The survey included a ‘trigger warning’ and a link to a webpage containing information about mental health resources. The study team had access to contact details of seed participants only (email address or phone number). The study was approved by the LSHTM Observational Research Ethics Committee (27901). We report our study against the Strengthening the Reporting of Observational Studies in Epidemiology (STROBE) checklist for cross-sectional studies [27].

### Survey questionnaire

The survey questionnaire (see **Supplementary Material**) included questions about familial mortality, and about the household wealth of the respondent’s closest family member living in Tigray. The survey was available in English and Tigrinya. Respondents could complete the survey themselves and on behalf of a spouse of Tigrayan descent. Respondents were asked to provide the age, or date of birth, and the status (i.e. deceased, alive, unknown) of each member of their immediate family including biological parents, biological siblings, and nieces/nephews. We requested the following information about deceased family members: (1) place/zone of death, (2) year/month of death, (3) age at deaths, and (4) primary cause of death. The questionnaire collected data elements consistent with the child survival and sibling survival modules of the Demographic Health Survey (DHS) questionnaire [28]. Respondents were also asked to provide information on household assets and infrastructure of their ‘closest family member still living in Tigray’. To minimise questionnaire response time we chose a subset of asset and infrastructure items (see **Supplementary Material**) included in the 2019 Ethiopia DHS Household Questionnaire [29].

### Statistical methods

Given the limited survey propagation below the initial seed level (see **Results**), we chose not to analyse the data as an RDS sample. Instead, we assumed a random sample of the Tigrayan diaspora and adjusted for potential selection bias relative to the population of Tigray by standardising the estimates for urban-rural residence and household wealth (see **Estimate Standardisation**). In the primary analysis, we excluded individuals who died outside Tigray.

#### Child mortality

We broadly followed the DHS method for estimating period-specific child mortality [28], using data on nieces/nephews. This method estimates mortality risk during a given secular period, and over an age span of interest, as one minus the product of probabilities of surviving each of several age strata within this span. Unlike the DHS method we used a single infant age stratum (0–11 months); calculated the exact fraction of each period that children in each age group spent at risk; and computed confidence intervals (CI) as the 95% percentile interval of 1000 bootstrap simulations of the dataset (instead of the jack-knife approach), with each simulation consisting of a random sample, with replacement, of all respondent identification numbers and their relatives (sized as per the number of respondents in the original dataset). We also estimated mortality amongst older children (5–14 years) and young adults (aged 15–24 years) using the same method: here the denominator is restricted to children who survived up to age five or age 15, respectively. We considered four periods of interest: January 2005-December 2009, January 2010-December 2014, January 2015-October 2020, and November 2020 (start of the conflict) to April 2023 (or the survey completion date, i.e. November 2022-April 2023). If month of death was not reported we assumed the midpoint of the period over which data were collected.

#### Adult and older adult mortality

We estimated adult mortality (15 to 49 years) based on sibling data by calculating a ratio of deaths to person-time that each individual contributed to a given period (see above), sex and age stratum (from 15 to 49 years, in increments of five years). The age range was chosen to align with the conventional age range for reporting adult mortality. We applied the same bootstrapping method (above) to compute 95% CIs for this cohort.

We also estimated mortality amongst older adults (using parent data) aged ≥ 60 years, as per the most commonly reported age-based definition of old age [30]. As deaths were more frequent in this age group, we extended the pre-war-periods to 1980–1989, 1990–1999, 2000–2009, and 2010 to October 2020.

#### Fertility patterns

To explore the validity of our data we computed several indicators of fertility using the DHS method [28]. We estimated period- and age-specific fertility rates (ASFR) using five-year age strata from 15 to 49 years, number of births (as the numerator), and person-time exposure amongst siblings as the denominator. For completeness, we report ASFR amongst female as well as male siblings, though the former is easier to interpret (relating only to woman’s biological children), and is more commonly measured. We also estimated period-specific total fertility rates as the sum of ASFR over all age strata multiplied by five (the width, in years, of each age stratum): this indicator expresses the expected number of children that an average person would have over their reproductive period 15 to 49 years. Lastly, we analysed the distribution of birth intervals by period, excluding first-born children. We compared our age-specific fertility rates with national DHS data [31], period-specific fertility rates with historical fertility rates for Tigray [32], and birth intervals with 2019 DHS data for Tigray [29].

#### Estimate standardisation

To assess and reduce selection bias due to differences in socio-economic status between the diaspora sample and the general population of Tigray, we constructed a wealth index (for rural and urban residents) as follows. First, we resolved missingness in some of the items through multivariate imputation by chain equations (MICE) (https://www.jstatsoft.org/article/view/v045i03) using random forest models trained on the entire non-missing respondent dataset, with five chains of 20 iterations each.

We combined data from our survey and the 2019 DHS (Tigray region data) and split the combined dataset into an urban and rural stratum. For each stratum, we used the ‘psych’ package in R (https://cran.r-project.org/web/packages/psych/citation.html) to generate a wealth index through principal components analysis with variance-covariance matrix decomposition, varimax-rotated factor loadings, and up to three principal components. We standardised all estimates by stratifying the dataset by urban-rural residence and wealth index quintile and attributing to each stratum a survey weight equal to the proportion of DHS 2019 observations falling within that stratum, assuming that the DHS was representative of Tigray’s population. We present both unstandardised and standardised estimates. See **Supplementary Material** for distributions of urban versus rural residence and wealth index quintiles.

#### Sensitivity analyses

We carried out two sensitivity analyses of the dataset. First, we excluded all observations related to the respondent’s spouse. Second, we assumed that a varying proportion of individuals reported to be still alive (about whom we only requested age) may have left Tigray prior to the survey date. For each assumed proportion, we repeated the analysis over 1000 simulations, with each simulation randomly selecting which individuals departed, and their age at departure.

### Patient public involvement in research

Members of the Tigrayan diaspora community were consulted in the conceptualisation and design of the study to ensure the accuracy, and appropriateness of the survey. A member of the Tigrayan diaspora community was a part of the study team, and facilitated this consultation process. Members of the diaspora community were directly involved in survey propagation by sharing the survey with up to five members of their diaspora peer networks. Key community members and organisations were also involved in outreach within the community to promote study awareness and participation, and to encourage survey completion. After the end of survey data collection, select study participants were asked to participate in a feedback session in which their experiences participating in the survey were explored [data not shown]. Community organisations, media, key community leaders, and other members of the diaspora will be involved in the dissemination of study results.

## Results

### Survey attrition

We chose to stop the survey after 1000 survey accesses (ultimately stopping at 1051) as we presumed near-saturation within the active networks (recruitment had slowed, and the number of ineligible respondents had increased primarily due to having a close family member previously complete the survey). Of the 1051 survey responses, 791 were analysable, of which almost nine in ten were from seed respondents, with limited propagation beyond this first survey generation (Table 1).

Of the 791 respondents, 162 also provided information on behalf of a spouse; thus, our sample includes data from 10,725 individuals (9,093 relatives of a respondent and 1,632 relatives of a spouse) from 953 families. The parent sub-sample includes 1906 individuals (17.8%), the sibling sub-sample 4,046 (37.7%) individuals, and the niece/nephew sub-sample 4,773 (44.5%). The sex distribution of family members was unremarkable.

**Table 1:** Survey eligibility, attrition, and propagation

	GROUP	RESPONDENT #	%
	ALL SURVEY ACCESSES	1051	100.0%
ELIGIBILITY	Outside age inclusion criteria	114/1051	10.8%
	Not a member of the diaspora	18/1051	1.7%
	Belongs to the same family as that of another respondent	79/1051	7.5%
	**ELIGIBLE**	**840/1051**	**79.9%**
ATTRITION	Did not consent	31/840	3.7%
	Consented but did not complete the survey	18/840	2.2%
	**COMPLETED THE SURVEY (ANALYSABLE)**	**791/840**	**94.2%**
PROPAGATION	1st generation (seed)	699/791	88.4%
	2nd generation	74/791	9.3%
	3rd generation	14/791	1.8%
	4th generation	4/791	0.5%

### Vital status and cause of death

**Fig. 1:**
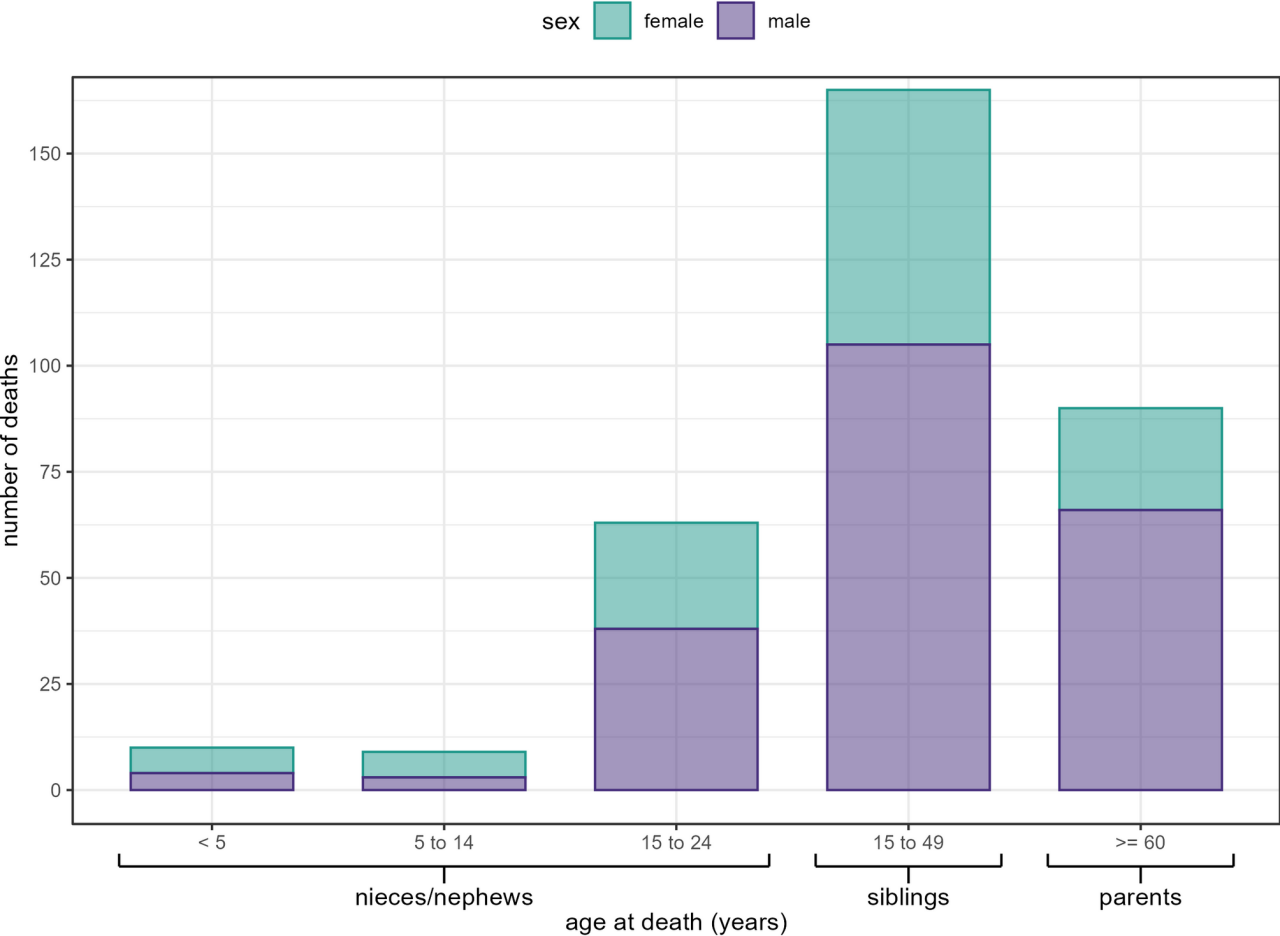
Number of deaths in Tigray during the war-period, by sex and age

The status of individuals, by relationship to the respondent, is summarised in Table 2. Overall, 8,750/10,725 (81.6%) were reported to be alive, 1,011 (9.4%) were deceased, and 964 (9.0%) were of unknown status. Eight-hundred and ten (80.1%) deaths occurred within Tigray including 310 (38.3%) during the war-period (November 2020-April 2023), of which 134 (43.2%) occurred in Central Tigray, 61 (19.7%) in Eastern Tigray, 32 (10.3%) in the Southern/Southeastern Zone, 26 both in North Western Tigray (8.4%) and the Mekelle Special Zone (8.4%), 25 (8.1%) in Western Tigray, and six (1.9%) in unknown locations. Deaths during the war-period by age and sex are presented in Fig. 1.

**Fig. 2:**
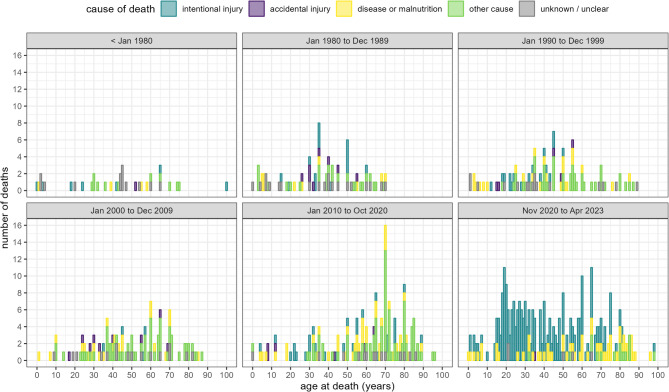
Age at death, by cause and period of death (for deaths in Tigray)

**Fig. 3:**
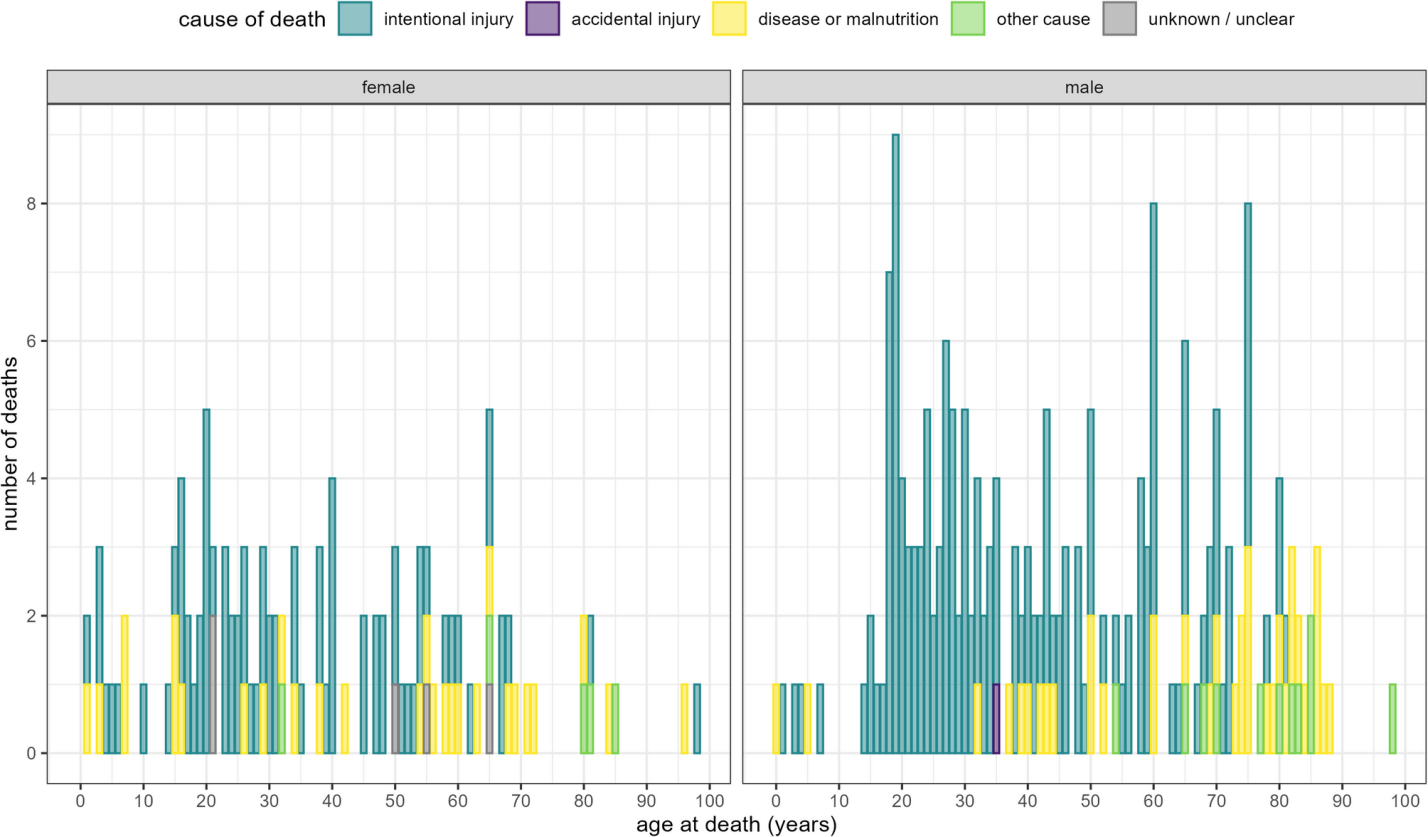
Age at death, by cause and sex (for deaths in Tigray)

Causes of death by period and sex are presented in Figs. 2 and 3 respectively. The most common causes of death during the war-period were intentional or war-related injury (*n* = 224; 72.3%) followed by starvation or disease (*n* = 64; 20.6%). Intentional injuries were by contrast uncommon during all pre-war-periods analysed. Of the 224 war-period intentional injury deaths, two-thirds were male (*n* = 149, 66.5%).

### Mortality estimates

**Fig. 4:**
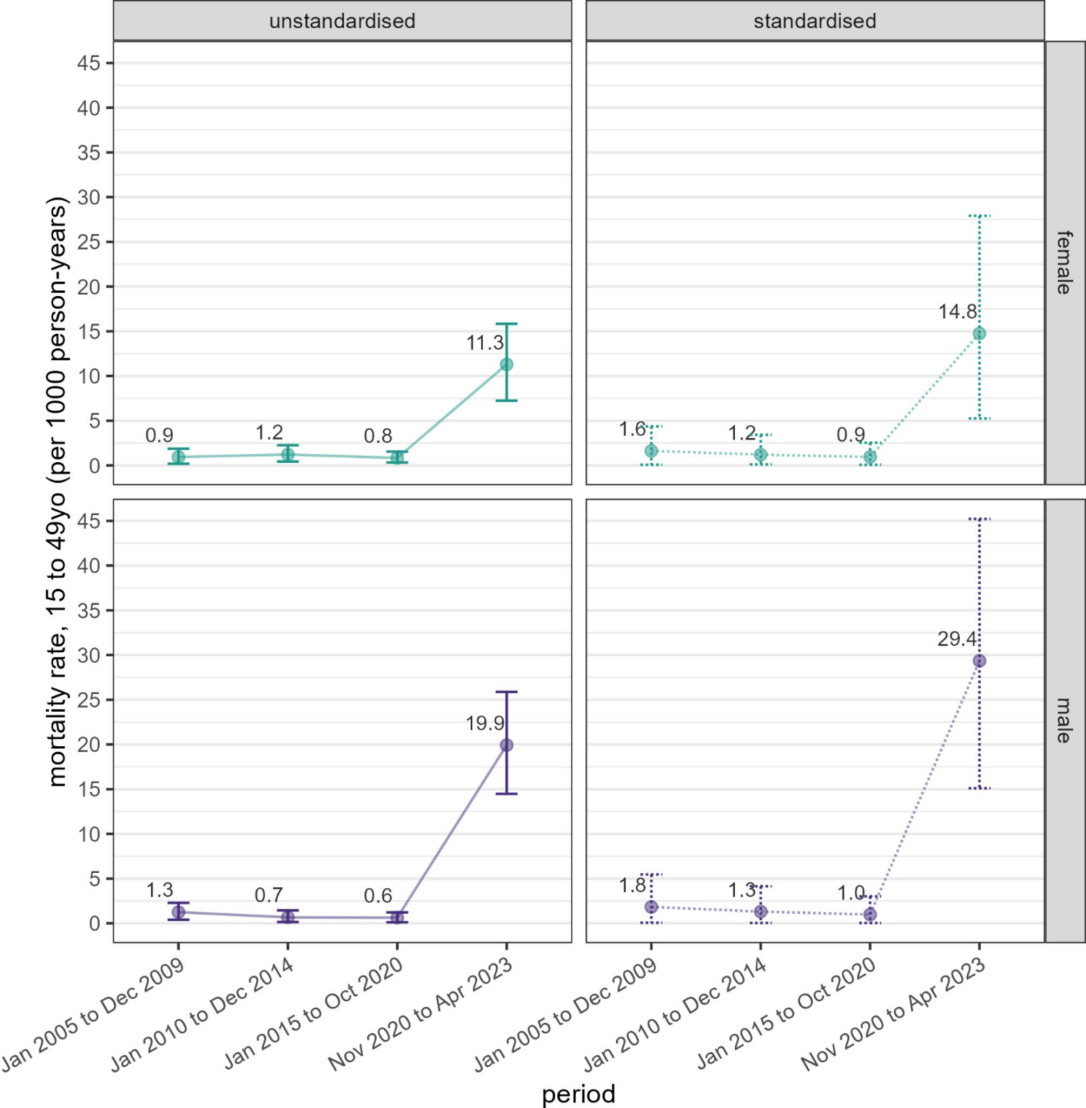
Standardised and unstandardised adult (15–49 years) mortality estimates, by period and sex. Points and labels indicate point estimates and vertical brackets 95% confidence intervals

Standardised and unstandardised child mortality estimates are shown in Table 3. We estimated a 32-fold increase in standardised mortality amongst children under five years during the war-period, relative to 2015–2020. Contrary to expected patterns, mortality reduced to 23.9 deaths per 1000 amongst children aged 5–14 years, and increased to 226 amongst young adults (15–24 years). For periods before the war estimated mortality was generally low or nil (see **Discussion**). Standardised and unstandardised mortality rates for adults aged 15 to 49 years, by sex, are presented in Fig. 4; age-disaggregated rates are included in the **Supplementary Material**. The standardised mortality rate for all adults (aged 15 to 49 years) was 21.3 (95%CI 10.9–26.0) per 1000 person-years during the war-period, with male mortality about double that for females. Prior to the war adult mortality rates appeared to have been declining, dropping from 1.7 (95%CI 0.6-3.0), to 1.3 (95%CI 0.4–3.4), to 1.0 (95%CI 0.3–1.9) in the periods 2005–2009, 2010–2014, and 2015–2020 respectively. During the war-period nearly all (*n* = 145, 87.9%) of the 165 reported deaths in this age group were attributed to intentional injury; with intentional injury accounting for 92.4% (*n* = 97/105) of deaths amongst men, and 80% (*n* = 48/60) of deaths amongst women aged 15–49 years.

**Table 2:** Number and status of family members, by relation to the respondent (includes status of relatives outside of Tigray)

Relation and status	SIBLINGS		NIECES/NEPHEWS		PARENTS		ALL RELATIVES	
	#	%	#	%	#	%	#	%
**Relatives of respondent**	**3389/4046**	**83.8%**	**4122/4773**	**86.4%**	**1582/1906**	**83.0%**	**9093/10,725**	**84.8%**
*Alive*	2780/3389	82.0%	3630/4122	88.1%	1048/1582	66.2%	7458/9093	82.0%
*Deceased*	266/3389	7.8%	96/4122	2.3%	461/1582	29.1%	823/9093	9.1%
*Unknown*	343/3389	10.1%	396/4122	9.6%	73/1582	4.6%	812/9093	8.9%
**Relatives of respondent’s spouse**	**657/4046**	**16.2%**	**651/4773**	**13.6%**	**324/1906**	**17.0%**	**1632/10,725**	**15.2%**
*Alive*	531/657	80.8%	572/651	87.9%	189/324	58.3%	1292/1632	79.2%
*Deceased*	57/657	8.7%	17/651	2.6%	114/324	35.2%	188/1632	11.5%
*Unknown*	69/657	10.5%	62/651	9.5%	21/324	6.5%	152/1632	9.3%
**TOTAL**	**4046/10,725**	**37.7%**	**4773/10,725**	**44.5%**	**1906/10,725**	**17.8%**	**10,725**	**100.0%**

**Table 3:** Standardised and unstandardised estimates of child mortality, by period and age stratum

AGE CATEGORY	PERIOD	UNSTANDARDISED ESTIMATE (95%CI)	STANDARDISED ESTIMATE (95%CI)
UNDER 5 YEAR MORTALITY (PER 1000 LIVE BIRTHS)	Jan 2005 to Dec 2009	0 (0 to 0)	0 (0 to 0)
	Jan 2010 to Dec 2014	0 (0 to 0)	0 (0 to 0)
	Jan 2015 to Oct 2020	2.8 (0 to 6.3)	1.0 (0 to 3.1)
	**Nov 2020 to Apr 2023**	**14.7 (4.1 to 28.1)**	**32.2 (1.9 to 74.7)**
5 TO 14 YEAR MORTALITY (PER 1000 CHILDREN REACHING AGE 5Y)	Jan 2005 to Dec 2009	0 (0 to 0)	0 (0 to 0)
	Jan 2010 to Dec 2014	2.2 (0 to 7.5)	2.0 (0 to 7.4)
	Jan 2015 to Oct 2020	3.4 (0 to 7.7)	7.2 (0 to 24.3)
	**Nov 2020 to Apr 2023**	**19.2 (4.6 to 38.4)**	**23.9 (0.3 to 65.6)**
15 TO 24 YEAR MORTALITY (PER 1000 CHILDREN REACHING AGE 15Y)	Jan 2005 to Dec 2009	0 (0 to 0)	0 (0 to 0)
	Jan 2010 to Dec 2014	0 (0 to 0)	0 (0 to 0)
	Jan 2015 to Oct 2020	1.3 (0 to 4.1)	0.1 (0 to 0.3)
	**Nov 2020 to Apr 2023**	**147.9 (93.9 to 207.3)**	**226.0 (106.7 to 343.4)**

**Fig. 5:**
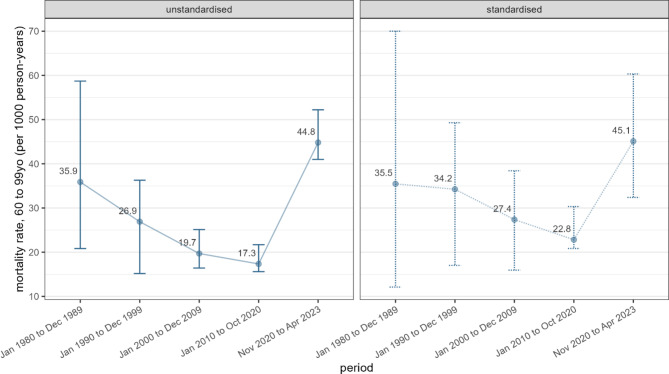
Standardised and unstandardised mortality rates in older adults (60–99 years), by period [Points and labels indicate point estimates and vertical brackets 95% confidence intervals]

The standardised mortality rate for older adults was 45.1 (95%CI 32.4–60.3) per 1000 person-years during the war-period (Fig. 5). As with adults, the mortality rate in older adults declined across the pre-war-periods, dropping from 35.5 (95%CI 12.1–70.0), to 34.2 (95%CI 17.0-49.3), to 27.4 (95%CI 15.9–38.4), to 22.8 (95%CI 20.8–30.3) in the periods 1980–1989, 1990–1999, 2000–2009, and 2010–2020 respectively. The mortality rate for older men (58.4 [95%CI 40.1–74.9]) was nearly twice that of older women (29.1 [95%CI 20.1–56.1]) during the war-period; however, mortality rates were comparatively high for older men across all pre-war-periods [data not shown].

The results of the sensitivity analyses (i.e. excluding individuals related to respondents’ spouse, and accounting for out-migration prior to the survey date) show minimal deviance from the primary analysis (**Supplementary Material**).

### Fertility patterns

Standardised and unstandardised period- and age-specific fertility rates and birth interval distributions are included in the **Supplementary Material**. Graphical comparison of ASFR between our sample and historical data from 2000, 2005, and 2011 for Ethiopia indicate comparatively lower fertility in younger age groups in our sample of female siblings of respondents, with ASFR peaking in the 30–34 year age bracket in our sample compared to a 25–29 year peak amongst the DHS population [31]. Female siblings of respondents’ spouses show a similar pattern of ASFR when compared to the DHS population over the same period [31]. Comparing period-specific fertility rates for Tigray [32] to standardised rates for respondents’ female siblings suggests lower fertility rates in our sample; however, the difference is less pronounced in earlier time periods. Standardised fertility rates for respondent’s male siblings are also lower than total fertility rates for Tigray [32]. Comparison of birth spacing with DHS data for Tigray [29] suggests similar birth spacing amongst both male and female siblings.

## Discussion

Our findings suggest a substantial increase in mortality amongst children, adults, and older adults in Tigray during the war-period when compared to pre-war-periods. As ours is the first study estimating mortality rates for all of Tigray, direct comparisons with the few other available studies are not possible; however, these other studies similarly evidence a sharp increase in mortality during the war-period. A large cross-sectional household survey carried out between October 2020 and May 2022 yielded estimates of the maternal mortality ratio (840 maternal deaths per 100,000 live births) [21], and the neonatal mortality rate (28.2 neonatal deaths per live births) [22]. Whilst these findings suggest a 3–4 fold increase in maternal mortality, and a 2–3 fold increase in neonatal mortality compared to pre-war estimates, they are likely to be under-estimates as the survey did not include the most conflict-affected regions of Tigray [22]. A separate analysis of the same sample estimated an U5MR of 59 per 1000 live births, suggesting a doubling compared to the pre-war-period [20]. In comparison, our standardised U5MR is lower (32.2), despite the fact that our sample captured all regions of Tigray, albeit with unknown relative coverage. Our pre-war U5MRs are implausibly low: for example, the Global Burden of Disease Study estimated a U5MR for Tigray of 33.1 in 2019 [33] whilst the 2019 DHS reports a U5MR of 43 [29]. Our estimates for older children and adolescents, whilst potentially also subject to some underestimation, suggest extremely elevated mortality in the war-period in what should otherwise be age groups with very low mortality rates.

We have greater confidence in the mortality estimates generated using our sibling data subset (see **Limitations**); yet, as with our niece/nephew subset, our pre-war mortality figures are implausibly low. However, comparing our standardised mortality estimate (21.30 [95%CI 10.92–26.05] for all adults during the war-period to the 2019 age-standardised, all-cause adult mortality rate (5.24) from the Global Burden of Disease Study for Tigray suggests a four-fold increase in adult mortality during the war-period [34]. The pre-war mortality rate amongst adults and older adults in our sample declined across all pre-war-periods. This decline was also observed in the Tigray Burden of Disease study [34] and the 2016 Demographic and Health Survey for Ethiopia [35], suggesting that the current war has undone decades of progress towards improved health. Nearly all deaths amongst adults during the war-period were attributed to intentional injury (87.9%). The standardised mortality rate in *older adults* in the current war-period (45.1 [95%CI 32.4–60.3]) is higher than the mortality rate (35.5 [95%CI 12.1–70.0]) for the period encompassing most of the Ethiopian Civil War (1974–1991), and the Tigray and Wollo Famine (1984–1985).

During the war-period 92.4% of deaths amongst men and 80% amongst women were attributed to intentional injury. Though men had doubled mortality compared to women, this ratio is typically higher in conflict settings (e.g [36, 37]). There is some evidence that ethnically-based conflict results in relatively high violent mortality amongst women [38]. Despite the conclusion by the UN’s ICHREE that extrajudicial killing in Tigray was, “…distinctly characterised by undertones of androcide and overwhelmingly targeted at fighting-age civilian males of Tigrayan ethnicity ”, our findings are inconsistent with the mortality pattern we would expect to observe if the military objective was solely to target adult males, and instead point to widespread violence against women and others who would have been unlikely to pose a combatant threat (i.e. children, the elderly) [39, p. 5]. However, as noted above, maternal mortality was found to have increased significantly during the war-period [21].

The Armed Conflict Location & Event Data (ACLED) Project aims to collect, verify, and publish reports of fatalities (collected from traditional media, mortality reports, local partner reports, and social media) resulting from violent conflict and protest [40]. According to ACLED, 5,325 people died in Tigray (including 3, 151 civilians) as a consequence of direct combat or violence targeting civilians between 4 November 2020 and 2 November 2022 [17]. Using a similar method, the University of Ghent estimated that there were between 7,060 and 12, 330 ‘deadly victims’ in Tigray over the same period [19].

Based on our adult mortality rate estimates, a mid-period (2021) Tigrayan population of about 5.71 million, and age composition as per DHS and United Nations age-specific mortality rate projections for Ethiopia as a whole in 2019 [41], we may crudely estimate an excess death toll of 102,466 (95%CI 17,455 to 159,382) within the population aged ≥ 15 years; this excludes children and may be an under-estimate given that pre-war mortality was likely lower in Tigray than the Ethiopian average (e.g. U5MR in 2019 was 43 in Tigray, versus 59 for Ethiopia) [29]. Additional estimates produced by researchers at the University of Ghent suggest that 518,000 civilians (311,000–808,000) have died over the conflict period as a result of exposure to conflict, starvation, and lack of access to health care [42]. However, this approach to mortality estimation (i.e. using unsophisticated multiplicative models to combine cause-specific mortality projections) is methodologically problematic and has been subject to considerable criticism with respect to its use in estimating deaths in Gaza [43]. Our estimate of over 102,000 excess deaths in Tigray during the war-period is significantly lower yet similarly evidences extreme loss of life. Though there is little practical value in comparing the total numbers of people killed across conflicts, it is notable that reliable estimates of mortality in Gaza suggest a total of 54,837 adults (aged 18+) were killed in the first nine months of the conflict, compared to 102,466 adults (aged 15+) in Tigray over the war-period lasting 24 months [44].

### Limitations

Whilst the survey was designed as an RDS, it achieved very little propagation, for reasons we do not well understand, despite extensive socialisation efforts. We standardised estimates to render the sample closer in terms of representativity to the (pre-war) characteristics of Tigray’s population. As a result, we observed that the sample was highly skewed towards urban and higher-wealth strata (see **Supplementary Material**). It is nonetheless possible that additional, unaccounted-for selection bias affected our estimates: for example, respondents may have originated disproportionately from specific locations within Tigray. Our survey also does not capture the experience of refugees who left Tigray, or others who were displaced to elsewhere within Ethiopia.

Our estimates of child mortality are based on an implausibly small number of reported deaths. Though underreporting of < 5 deaths is a known problem in many settings (e.g [45, 46]) this phenomenon has been shown to increase considerably when less comprehensive data collection instruments are used [47]. The questionnaire’s flow may also have contributed to underreporting of deaths amongst nieces/nephews due to survey fatigue as these questions occurred at the end of the survey. It is also possible that respondents are simply less likely to have information about nieces/nephews compared to parents/siblings, or are otherwise unwilling to share details of child deaths, e.g. to avoid potential stigma. We did not collect information on the extent to which respondents felt knowledgeable about the status of their family in Tigray.

Feedback from our study using webRDS for estimating mortality in Yemen suggests that some seed respondents with large families found the survey too time consuming [25]. Respondents were provided with information about the purpose of the survey which may have led to under-reporting of deaths not directly caused by conflict. Finally, we incorporated few validation rules into the mortality survey to avoid frustrating participants, and possibly leading them to abandon the survey (particularly for those with large families). We made minor adjustments to account for obvious input errors which may have led to some misclassification. However, the number of input errors was low.

## Conclusions

Political tensions in Tigray persist. The lack of clear provision for the protection of civilians, the failure to address the underlying causes of the Tigray conflict, the absence of evidence of meaningful political dialogue, and reports of ongoing attacks against civilians in Tigray, suggest that renewal of hostilities in Tigray may be imminent [5, 6]. There has been little visible progress towards acting on the provisions set out in the Cessation of Hostilities Agreement [48]. Our findings evidence extreme loss of life during the recent conflict in Tigray, and a reversal of decades of progress towards reduced premature mortality. We encourage relevant actors to consider our findings in light of a stagnant peace process and the prospect of another war in Tigray.

## Electronic supplementary material

Below is the link to the electronic supplementary material.

Supplementary Material 1

Supplementary Material 2

Supplementary Material 3

Supplementary Material 4

Supplementary Material 5

Supplementary Material 6

Supplementary Material 7

## Data Availability

Data are available from the corresponding author upon reasonable request.

## Abbreviations

ACLED: Armed Conflict Location & Event Data
ASFR: Age-specific fertility rates
CI: Confidence interval
CMR: Crude mortality rate
DHS: Demographic health survey
ICHREE: International Commission of Human Rights Experts on Ethiopia
IDP: Internally displaced persons
MICE: Multivariate imputation by chain equations
MMR: Maternal mortality ratio
NMR: Neonatal mortality ratio
OR: Odds ratio
ODK: ODK (formerly Open Data Kit)
RDS: Respondent-driven sampling
TPLF: Tigray People’s Liberation Front
U5MR: Under five mortality rate
UNHRC: UN Human Rights Council
webRDS: Web-based respondent-driven sampling
