# Supplementary material for: Conflict-attributable mortality in Tigray Region, Ethiopia: Evidence from a survey of the Tigrayan diaspora
Source: Popul Health Metr. 2025 May 22;23:19. doi: 10.1186/s12963-025-00380-2 (PMC12096794; doi:10.1186/s12963-025-00380-2)
Supplement: Supplementary file 1 — Supplementary Material 1 [file 12963_2025_380_MOESM1_ESM.docx]

**SUPLEMENTARY MATERIAL #1**

# ELIGIBILITY

**You can participate in the study by completing this survey. Before we begin the survey we will need to determine if you are eligible to participate in this study.**

Please help us by answering the following questions:

Are you between 18 and 49 years of age?

Are you currently living outside of Tigray?

Do you think that a member of your close family (e.g. parents, spouse, brothers and sisters, nieces and nephews) has already completed this survey?

# CONSENT

**Participation in the study is voluntary. It is important that you consent freely to participate in the study. Please carefully read through the following statements:**

I understand that I will not be asked for any information that could identify me or anyone in my family. I understand that the data from this study will be published but that no one will be able to identify me or anyone in my family from the published data.

I understand that at the end of the survey I may be asked to invite others in my social network to also take this survey.

I consent to participate in the study by completing this survey.

# TRIGGER

**This survey will ask you questions about the loss of your loved ones in Tigray. We understand that this topic may be distressing. Please remember that your mental health should always come first. We understand if you choose not to continue with the survey. If you do wish to continue please accept our gratitude for taking the time to complete this survey, your contribution will make a difference.**
Do you wish to continue with the survey?

# BORN

Were you born in Tigray?

Please tell us in which zone you were living before you left Tigray.

# SES

**NOTE: If you have several close family members still living in Tigray please think of the one closest to you in age (and their household) when answering the questions below.**

We are going to ask you some questions about your family members who remain in Tigray. Your answers to these questions will help us to determine how representative our study participants are when compared to the population in Tigray.

Please answer the following questions about how to best describe your closest family member still living in Tigray.

Before the start of the current conflict in Tigray, was your closest family member in Tigray living in a urban or rural area?

Before the start of the current conflict in Tigray, which of these items did your closest family member in Tigray have access to?

Before the start of the current conflict in Tigray, which types of transportation did your closest family member in Tigray have access to?

Before the start of the current conflict in Tigray, what was the primary source of drinking water for your closest family member in Tigray?

Before the start of the current conflict in Tigray, which type of sanitation facilities did your closest family member in Tigray have access to?

Were these sanitation facilities shared with another family?

Before the start of the current conflict in Tigray, which type of flooring did your closest family member in Tigray have in his/her home?

# MOTHER

**NOTE: By this we mean your biological mother.**

We would like to ask you some questions about your mother.

Please tell us if your mother is alive or deceased.

What is your mother's current age?

Did your mother die in Tigray?

In what year did your mother die?

In what month did your mother die?

How old was your mother when she died?

In which zone was your mother living when she died?

What was the main cause of your mother's death?

Do you believe that this death could have been prevented if health services in Tigray had not been disrupted by the current conflict (including the blockade)?

# FATHER

We would like to ask you some questions about your father.

Please tell us if your father is alive or deceased.

What is your father's current age?

Did your father die in Tigray?

In what year did your father die?

In what month did your father die?

How old was your father when he died?

In which zone was your father living when he died?

What was the main cause of your father's death?

Do you believe that this death could have been prevented if health services in Tigray had not been disrupted by the current conflict (including the blockade)?

# SISTERS

**NOTE: By this we mean your sisters born to the same mother as you. Also included are sisters who were born to your mother, but are not the biological daughters of your father. We would like you to include any sisters who may have died soon after being born including any baby who cried, who made any movement, sound, or effort to breathe, or who showed any other signs of life even for a very short time.**

We would like to ask you about your biological sisters from the same mother.

Please tell us how many sisters you have (alive or deceased).

Please tell us if your {NUMBER} sister is alive or deceased.

What is your {NUMBER} sister's current age?

Did your {NUMBER} sister die in Tigray?

In what year did your {NUMBER} sister die?

In what month did your {NUMBER} sister die?

How old was your {NUMBER} sister when she died?

In which zone was your {NUMBER} sister living when she died?

What was the main cause of your {NUMBER} sister's death?

Do you believe that this death could have been prevented if health services in Tigray had not been disrupted by the current conflict (including the blockade)?

Does your {NUMBER} sister have any biological children?

How many biological children does your {NUMBER} sister have?

# SISTER CHILDREN

**NOTE: This includes any children who may have died soon after being born including any baby who cried, who made any movement, sound, or effort to breathe, or who showed any other signs of life even for a very short time.**

Please tell us the gender of your {NUMBER} sister's {NUMBER} child.

Please tell us if your {NUMBER} sister's {NUMBER} child is alive or deceased.

What is your {NUMBER} sister's {NUMBER} child's current age?

Did your {NUMBER} sister's {NUMBER} child die in Tigray?

In what year did your {NUMBER} sister's {NUMBER} child die?

In what month did your {NUMBER} sister's {NUMBER} child die?

How old was your {NUMBER} sister's {NUMBER} child when she or he died?

In which zone did your {NUMBER} sister's {NUMBER} child die?

What was the main cause of your {NUMBER} sister's {NUMBER} child's death?

Do you believe that this death could have been prevented if health services in Tigray had not been disrupted by the current conflict (including the blockade)?

# BROTHERS

**NOTE: By this we mean your brothers born to the same mother as you. Also included are brothers who were born to your mother, but are not the biological sons of your father. We would like you to include any brothers who may have died soon after being born including any baby who cried, who made any movement, sound, or effort to breathe, or who showed any other signs of life even for a very short time.**

We would like to ask you about your biological brothers from the same mother.

Please tell us how many brothers you have (alive or deceased).

Please tell us if your {NUMBER} brother is alive or deceased.

What is your {NUMBER} brother's current age?

Did your {NUMBER} brother die in Tigray?

In what year did your {NUMBER} brother die?

In what month did your {NUMBER} brother die?

How old was your {NUMBER} brother when he died?

In which zone was your {NUMBER} brother living when he died?

What was the main cause of your {NUMBER} brother's death?

Do you believe that this death could have been prevented if health services in Tigray had not been disrupted by the current conflict (including the blockade)

Does your {NUMBER} brother have any biological children?

How many biological children does your {NUMBER} brother have?

# BROTHER CHILDREN

**NOTE: This includes any children who may have died soon after being born including any baby who cried, who made any movement, sound, or effort to breathe, or who showed any other signs of life even for a very short time.**

Please tell us the gender of your {NUMBER} brother's {NUMBER} child.

Please tell us if your {NUMBER} brother's {NUMBER} child is alive or deceased.

What is your {NUMBER} brother's {NUMBER} child's current age?

Did your {NUMBER} brother's {NUMBER} child die in Tigray?

In what year did your {NUMBER} brother's {NUMBER} child die?

In what month did your {NUMBER} brother's {NUMBER} child die?

How old was your {NUMBER} brother's {NUMBER} child when she or he died?

In which zone did your {NUMBER} brother's {NUMBER} child die?

What was the main cause of your {NUMBER} brother's {NUMBER} child's death?

Do you believe that this death could have been prevented if health services in Tigray had not been disrupted by the current conflict (including the blockade)?

# SPOUSE

Please tell us your marital status.

Is your current spouse (or were any previous spouses) of Tigrayan background?

We would like to ask you some questions about your spouse's family. In case there is more than one marriage, it does not matter which spouse you choose but we would recommend that you choose the spouse whose family is the most familiar to you.

Would you be willing to answer questions about your spouse's family?

How many spouses of Tigrayan background have you had (including your current spouse)?

Please tell us which spouse you will be answering questions about.

# NETWORK SIZE

We would now like to ask you to estimate the size of your Tigrayan social network. We will use this information to determine how representative our data are.

Your network size is an estimate of the number of people you know, and who know you. These people must:

✔ **Be of Tigrayan background.**

✔ **Be living outside of Tigray (please do not count people who are currently living in Tigray).**

✔ **Be people you have interacted with (either in person or online) at least once in the past 12 months.**

Please tell us how many people are in your Tigrayan social network.
