# Supplementary material for: Conflict-attributable mortality in Tigray Region, Ethiopia: Evidence from a survey of the Tigrayan diaspora
Source: Popul Health Metr. 2025 May 22;23:19. doi: 10.1186/s12963-025-00380-2 (PMC12096794; doi:10.1186/s12963-025-00380-2)
Supplement: Supplementary file 2 — Supplementary Material 2 [file 12963_2025_380_MOESM2_ESM.docx]

**SUPLEMENTARY MATERIAL #2**

Wealth index items, distribution of urban vs rural residence, distribution of wealth index quintiles

## Wealth index construction

Questionnaire items used to construct a socio-economic wealth index. To avoid data sparsity, some items were grouped into a single category before constructing the wealth index.

| **Item category** | | **Items** | | |
| --- | --- | --- | --- | --- |
| Assets | | Electricity, radio, television, telephone (fixed), telephone (mobile), refrigerator, watch, computer, bank account, table, chair, bed (cotton, with springs), electric mitad (bread-making grill), kerosene pressure lamp | | |
| Transportation means | | Bicycle, motorcycle or scooter, car or truck, cart, bajaj (minivan) | | |
| Sanitation sharing | | Whether sanitation source is shared or single-household | | |
| Sanitation type | | **DHS survey item** | **RDS survey item** | **Harmonised item** |
|  |  | flush to piped sewer system | flush elsewhere | flush elsewhere |
|  |  | flush to septic tank | flush to septic tank | flush to septic tank |
|  |  | flush to pit latrine | flush to pit latrine | flush to pit latrine |
|  |  | flush to somewhere else | flush elsewhere | flush elsewhere |
|  |  | flush, don't know where | flush elsewhere | flush elsewhere |
|  |  | ventilated improved pit latrine | other type of toilet | other type of toilet |
|  |  | pit latrine with slab | pit latrine with slab | pit latrine with slab |
|  |  | pit latrine without slab/open pit | pit latrine without slab/open pit | pit latrine without slab |
|  |  | no facility/bush/field | no facility / bush/ field | no facility / bush/ field |
|  |  | composting toilet | other type of toilet | other type of toilet |
|  |  | bucket toilet | other type of toilet | other type of toilet |
|  |  | hanging toilet/latrine | hanging toilet / latrine | hanging toilet / latrine |
|  |  | other | other type of toilet | other type of toilet |
| **Item category** | **Group** | **DHS survey item** | **RDS survey item** | **Harmonised item** |
| Water source | 1 | cart with small tank | cart with small tank | cart with small tank |
|  |  | rainwater | rainwater | rainwater |
|  |  | tanker truck | tanker truck | tanker truck |
|  |  | other | other | other |
|  | 2 | piped to yard/plot | piped to yard/plot | piped to yard/plot |
|  |  | piped to neighbor | piped to neighbor | piped to neighbor |
|  | 3 | tube well or borehole | tube well or borehole | tube well or borehole |
|  |  | protected well | protected well | protected well |
|  |  | protected spring | protected spring | protected spring |
|  | 4 | unprotected spring | unprotected spring | unprotected spring |
|  |  | unprotected well | unprotected well | unprotected well |
|  |  | river/dam/lake/ponds/stream/canal/irrigation channel | surface | surface |
|  | 5 | piped into dwelling | piped into dwelling | piped into dwelling |
|  |  | bottled water | bottled water | bottled water |
|  | 6 | public tap/standpipe | public tap/standpipe | public tap/standpipe |
| Dwelling floor type | 1 | earth/sand | earth/sand | earth/sand |
|  |  | dung | dung | dung |
|  |  | wood planks | wood planks | wood planks |
|  |  | palm/bamboo | palm/bamboo | palm/bamboo |
|  |  | carpet | carpet | carpet |
|  | 2 | vinyl or asphalt strips | vinyl or asphalt strips | vinyl or asphalt strips |
|  |  | ceramic tiles | ceramic tiles | ceramic tiles |
|  |  | cement | cement | cement |
|  |  | n/a | other floor type | other floor type |


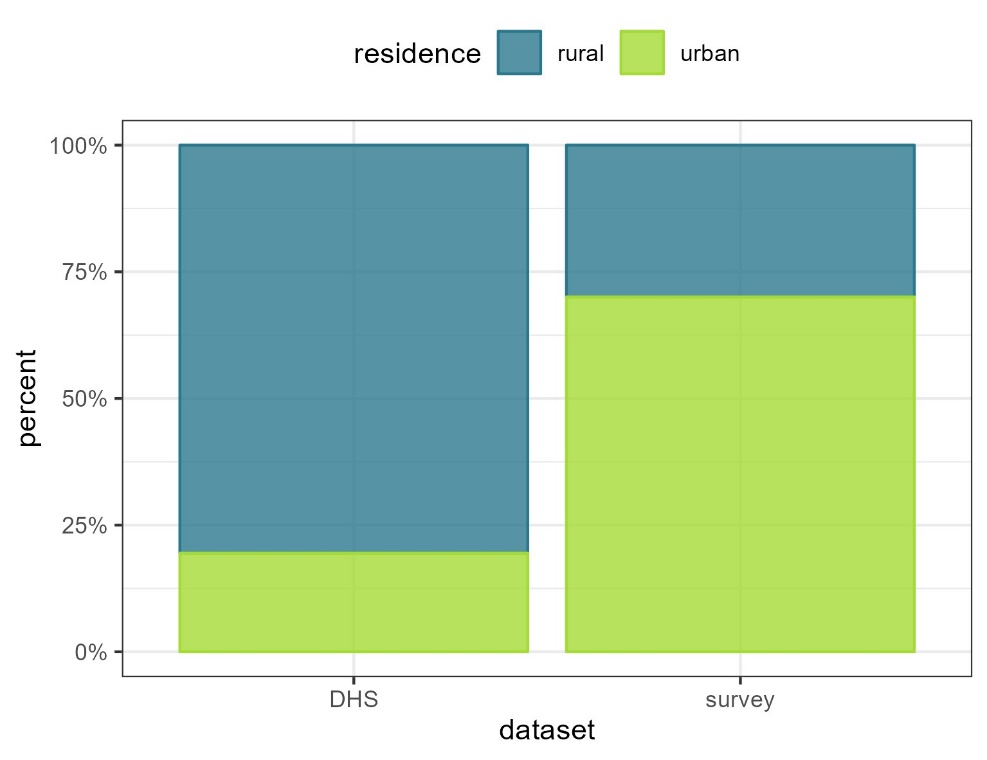


Distribution of urban versus rural residence, by source dataset (DHS 2019 versus this survey).

**
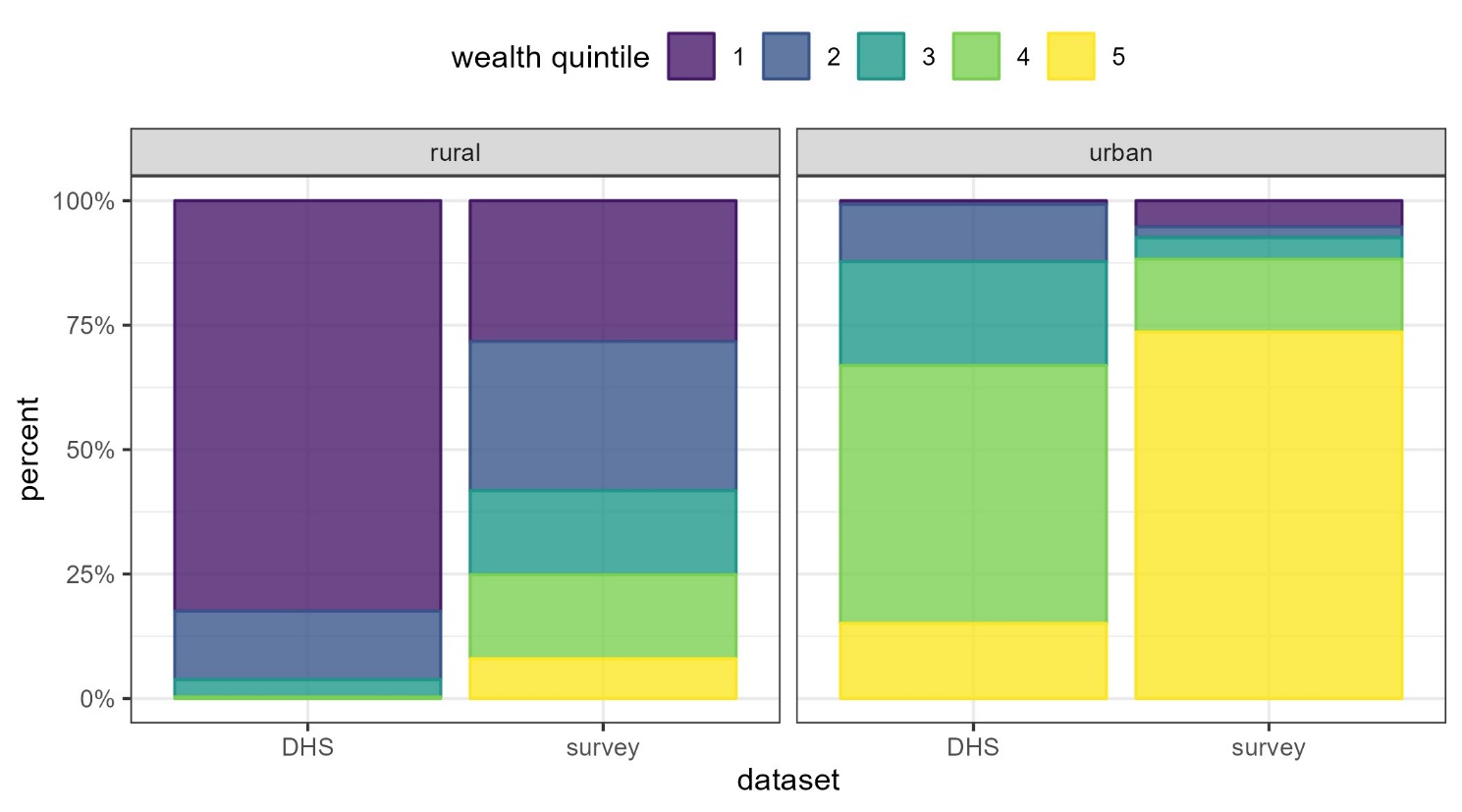
**

Distribution of wealth index quintiles, by urban versus rural residence and source dataset (DHS 2019 versus this survey).
