# Supplementary material for: Conflict-attributable mortality in Tigray Region, Ethiopia: Evidence from a survey of the Tigrayan diaspora
Source: Popul Health Metr. 2025 May 22;23:19. doi: 10.1186/s12963-025-00380-2 (PMC12096794; doi:10.1186/s12963-025-00380-2)
Supplement: Supplementary file 4 — Supplementary Material 4 [file 12963_2025_380_MOESM4_ESM.docx]

**SUPPLEMENTARY MATERIALS #4**

Standardised and unstandardised mortality (adults)


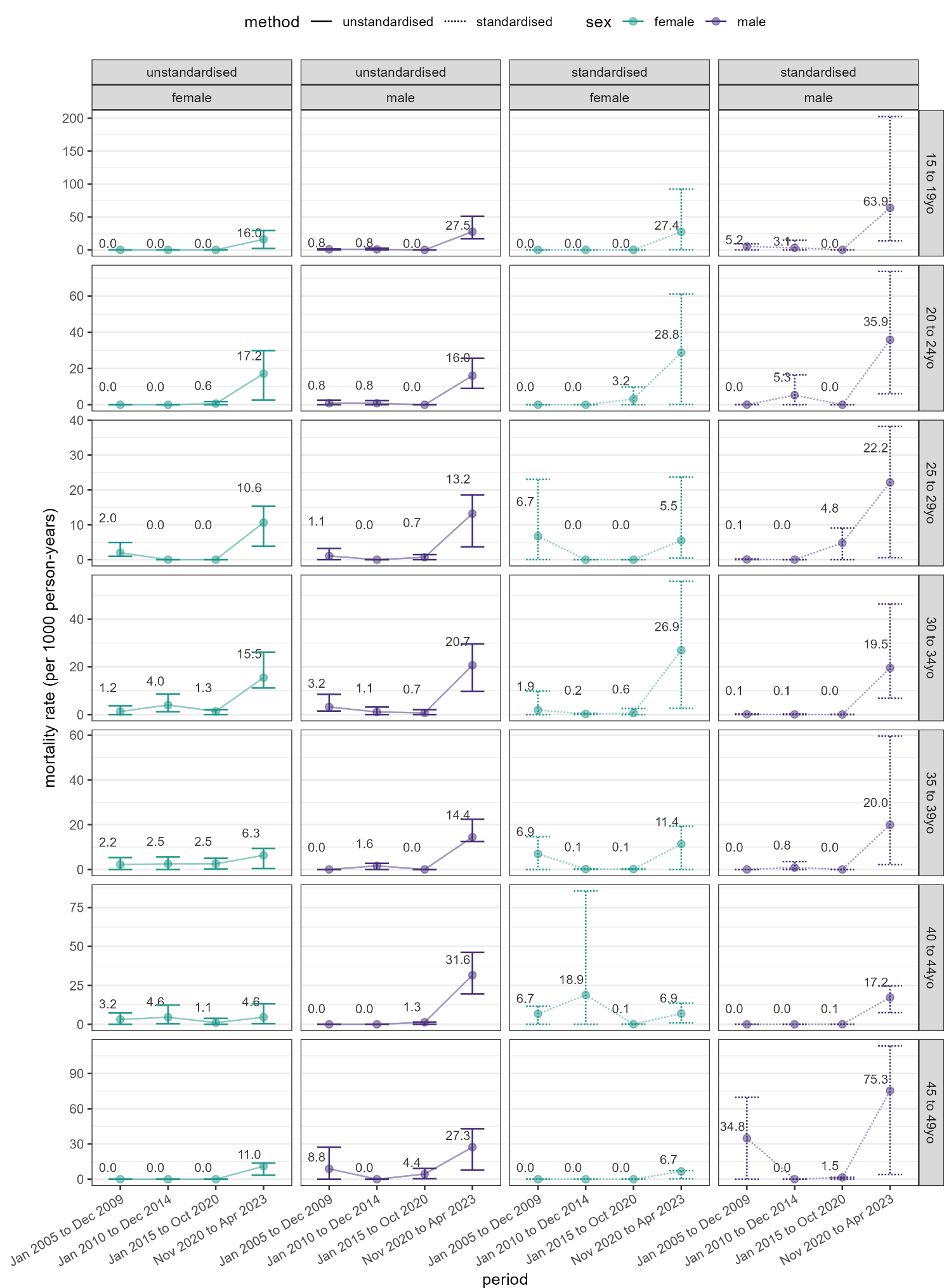


Unstandardised and standardized mortality rates among siblings of the respondents or their spouses, by period, sex and age stratum. Points and labels indicate point estimates, and vertical brackets 95%CIs.
