# Supplementary material for: Conflict-attributable mortality in Tigray Region, Ethiopia: Evidence from a survey of the Tigrayan diaspora
Source: Popul Health Metr. 2025 May 22;23:19. doi: 10.1186/s12963-025-00380-2 (PMC12096794; doi:10.1186/s12963-025-00380-2)
Supplement: Supplementary file 5 — Supplementary Material 5 [file 12963_2025_380_MOESM5_ESM.docx]

**SUPPLEMENTARY MATERIALS #5**

Sensitivity analysis

Table 3. Unstandardised and standardized estimates of child mortality, by period and age stratum, based on sensitivity analysis 1 (relatives of the respondent’s spouse excluded from analysis).

| Indicator | Period | Unstandardised estimate (95%CI) | Standardised estimate (95%CI) |
| --- | --- | --- | --- |
| under 5yo mortality (per 1000 live births) | Jan 2005 to Dec 2009 | 0 (0 to 0) | 0 (0 to 0) |
|  | Jan 2010 to Dec 2014 | 0 (0 to 0) | 0 (0 to 0) |
|  | Jan 2015 to Oct 2020 | 3.2 (0 to 7.8) | 1.2 (0 to 3.5) |
|  | Nov 2020 to Apr 2023 | 12.2 (2.3 to 26.0) | 34.4 (2.2 to 79.2) |
| 5 to 14yo mortality (per 1000 children reaching age 5y) | Jan 2005 to Dec 2009 | 0 (0 to 0) | 0 (0 to 0) |
|  | Jan 2010 to Dec 2014 | 2.6 (0 to 7.9) | 2.2 (0 to 6.8) |
|  | Jan 2015 to Oct 2020 | 2.3 (0 to 6.2) | 8.0 (0 to 26.4) |
|  | Nov 2020 to Apr 2023 | 15.9 (3.2 to 30.2) | 22.4 (0.2 to 62.5) |
| 15 to 24yo mortality (per 1000 children reaching age 15y) | Jan 2005 to Dec 2009 | 0 (0 to 0) | 0 (0 to 0) |
|  | Jan 2010 to Dec 2014 | 0 (0 to 0) | 0 (0 to 0) |
|  | Jan 2015 to Oct 2020 | 1.4 (0 to 4.9) | 0.1 (0 to 0.3) |
|  | Nov 2020 to Apr 2023 | 139.9 (77.7 to 209.5) | 194.2 (67.5 to 346.8) |
