# Supplementary material for: Conflict-attributable mortality in Tigray Region, Ethiopia: Evidence from a survey of the Tigrayan diaspora
Source: Popul Health Metr. 2025 May 22;23:19. doi: 10.1186/s12963-025-00380-2 (PMC12096794; doi:10.1186/s12963-025-00380-2)
Supplement: Supplementary file 6 — Supplementary Material 6 [file 12963_2025_380_MOESM6_ESM.docx]

**SUPPLEMENTARY MATERIALS #6**


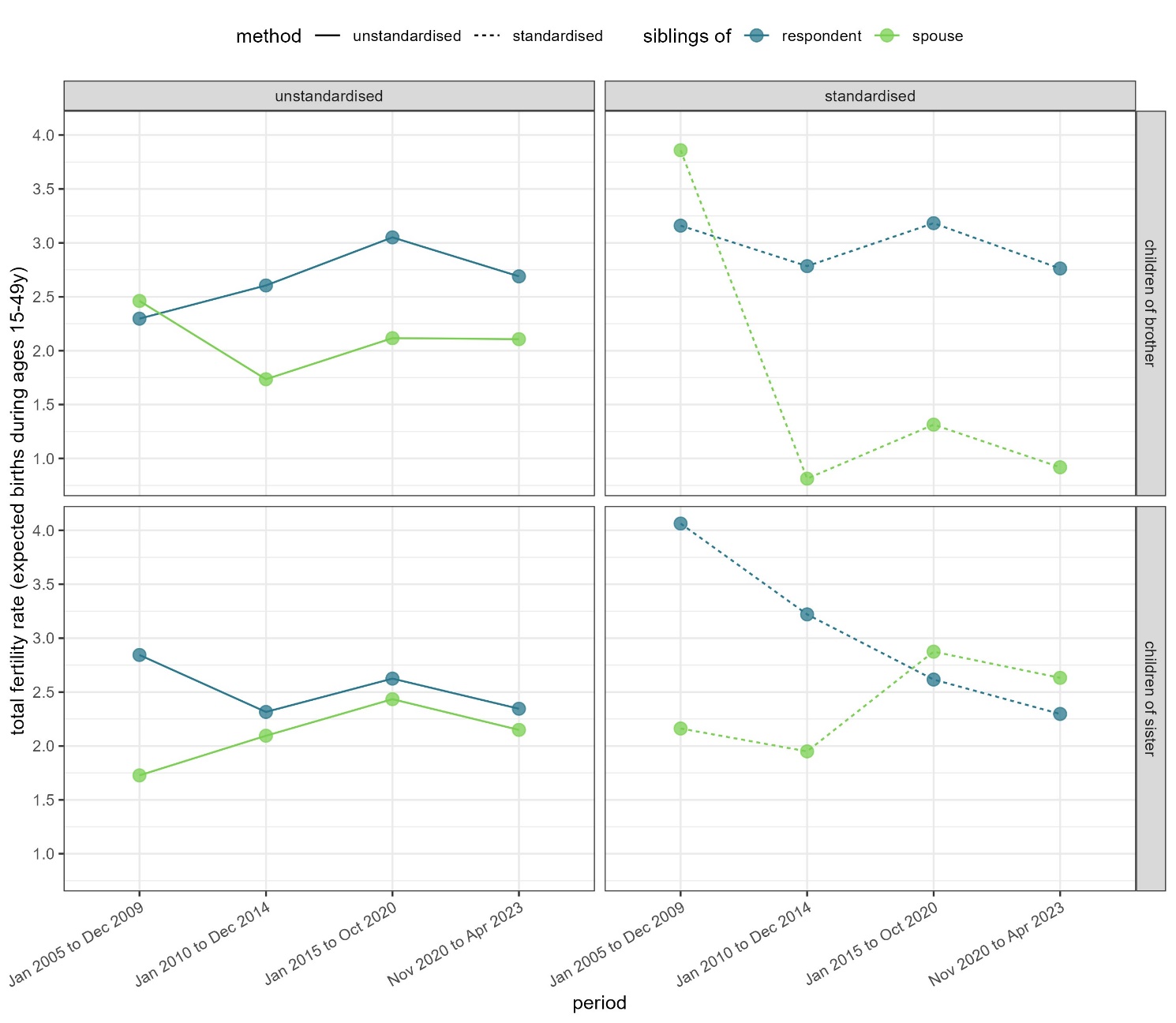


Estimated standardized and unstandardized total fertility rate, by period, parent’s sex and relation to the respondent.

Standardised and unstandardised fertility rates


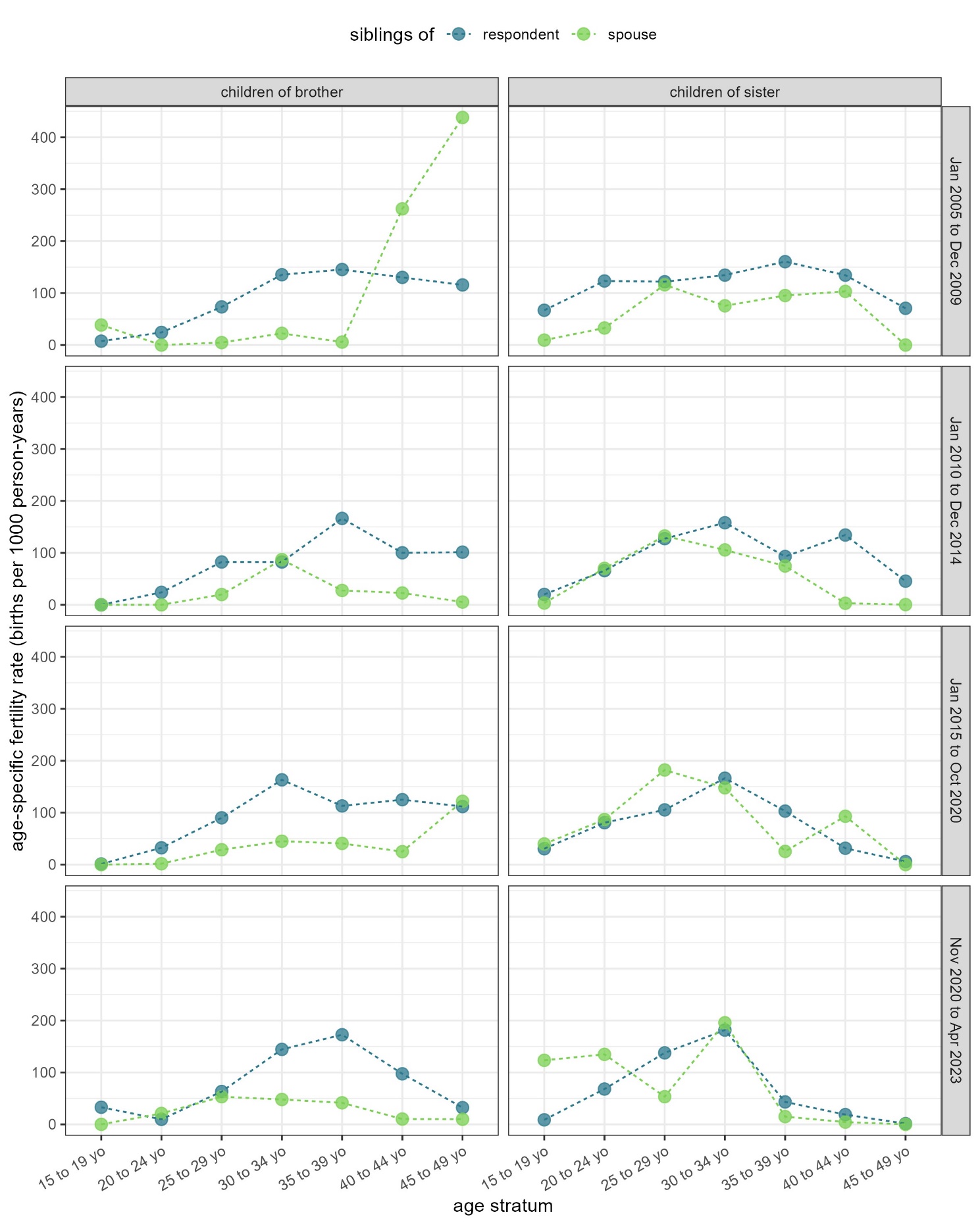


Standardised age-specific fertility rates by period, sex of the parent and relation to the respondent.


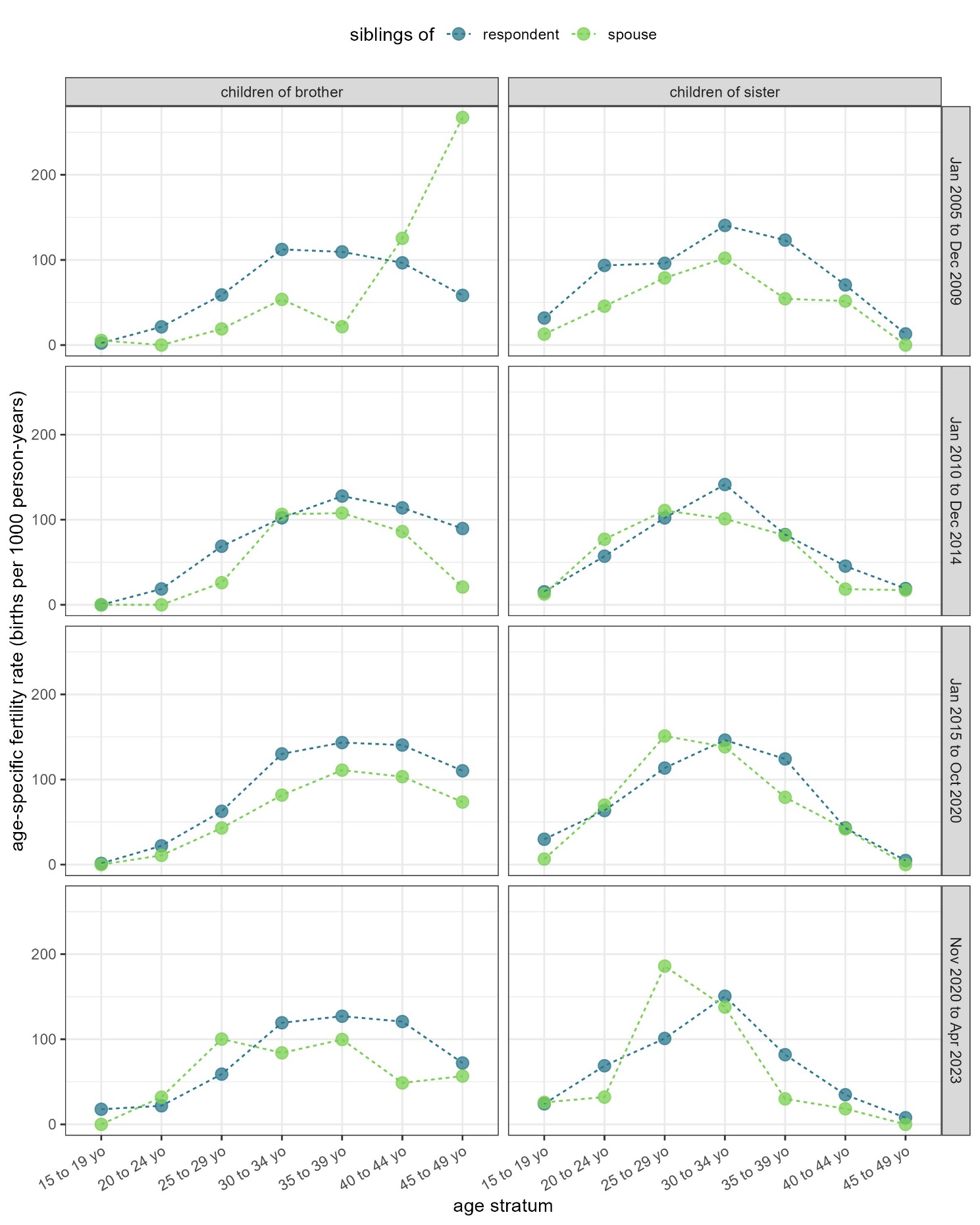


Unstandardised age-specific fertility rates by period, sex of the parent and relation to the respondent.

FC
