# Supplementary material for: Conflict-attributable mortality in Tigray Region, Ethiopia: Evidence from a survey of the Tigrayan diaspora
Source: Popul Health Metr. 2025 May 22;23:19. doi: 10.1186/s12963-025-00380-2 (PMC12096794; doi:10.1186/s12963-025-00380-2)
Supplement: Supplementary file 7 — Supplementary Material 7 [file 12963_2025_380_MOESM7_ESM.docx]

**SUPPLEMENTARY MATERIALS #7**

Birth interval


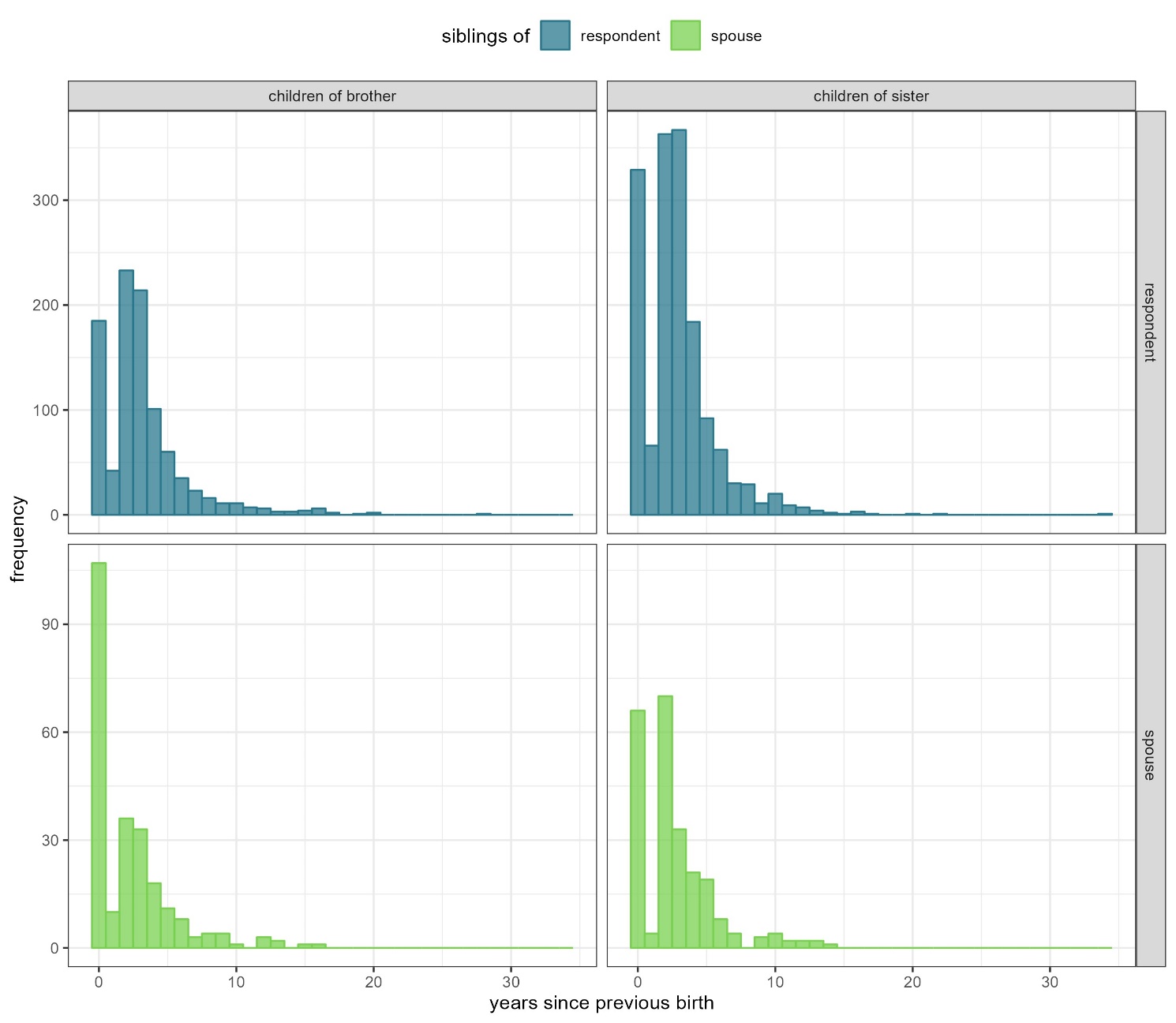


Distribution of birth intervals (excluding the first-born child) by parent’s sex and relation to the respondent.
